# The influence of physical exercise on adolescents’ externalizing problem behaviors: mediating effects of parent–child relationships, self-esteem, and self-control

**DOI:** 10.3389/fpubh.2024.1452574

**Published:** 2024-10-25

**Authors:** Long Cui, Yumei Xing, Jixing Gu, Hao Zhou, Lin Zhang, Yifeng Bu

**Affiliations:** ^1^Institute of Physical Education, Jiangsu Normal University, Xuzhou, China; ^2^Library, Jiangsu Normal University, Xuzhou, China

**Keywords:** physical exercise, externalizing problem behaviors, parent–child relationships, self-esteem, self-control, adolescents

## Abstract

**Background:**

Externalizing problem behaviors can significantly and negatively impact adolescents’ learning, daily life, and future socialization. While physical exercise is believed to inhibit adolescents’ externalizing problem behaviors, the extent of its effect and the mediating mechanisms remain unclear.

**Methods:**

This study is based on data from the 2018 China Family Panel Studies (CFPS). The CFPS project employed the Externalizing Problem Behaviors Scale (EPBS), the Rosenberg Self-Esteem Scale (RSES), and the Self-Control Scale (SCS) to measure adolescents’ externalizing problem behaviors, self-esteem, and self-control. Additionally, physical exercise was measured by collecting data on the time and frequency of adolescents’ physical exercise. Parent-child relationships were evaluated using a composite variable that included four dimensions: frequency of quarrels, frequency of heart-to-heart talks, parental awareness of their children’s whereabouts, and children’s trust in their parents. Based on data collection and variable construction, this study employed multiple linear regression, propensity score matching, and quantile regression to analyze the impact of physical exercise on adolescents’ externalizing problem behaviors and the heterogeneity of these effects. Additionally, the Bootstrap mediation effect test was employed to explore the mediating roles of parent-child relationships, self-esteem, and self-control in this process.

**Results:**

The analysis demonstrates that physical exercise significantly inhibits adolescents’ externalizing problem behaviors (*β* = −0.095, *p* < 0.01), although the effect varies significantly among different populations. Compared to males (*β* = −0.077, *p* < 0.1), rural residents (*β* = −0.065, *p* > 0.1), individuals with poorer family economic status (*β* = −0.080, *p* < 0.1), and those with more severe problem behaviors (*τ* = 0.75, *β* = −0.086, *p* < 0.05), physical exercise yields a more pronounced inhibitory effect on females (*β* = −0.113, *p* < 0.01), urban residents (*β* = −0.134, *p* < 0.01), individuals with better family economic status (*β* = −0.115, *p* < 0.01), and those with milder problem behaviors (*τ* = 0.25, *β* = −0.112, *p* < 0.01). Furthermore, through enhancements in parent-child relationships (CI: -0.015; -0.002), self-esteem (CI: -0.019; -0.003), and self-control (CI: -0.055; -0.025), physical exercise indirectly mitigates adolescents’ externalizing problem behaviors.

**Conclusion:**

Physical exercise significantly reduces adolescents’ externalizing problem behaviors, with effects differing across various groups. Parent-child relationships, self-esteem, and self-control mediate this relationship, underscoring the positive influence of exercise on adolescent behavior.

## 1 Introduction

Problem behaviors are abnormal behaviors that hinder individual social adaptation during personal development and are typically categorized into internalizing and externalizing problem behaviors (1). Internalizing problem behaviors involve responses to internal negative emotions, such as depression and anxiety (2), while externalizing problem behaviors refer to negative reactions to the external environment, such as indiscipline and aggression (3). Adolescent externalizing problem behaviors have received significant attention from scholars and practitioners in psychology, education, and sociology for their visible impact and prevalence during adolescence. This focus is justified by numerous studies showing that the transition from childhood to adolescence is characterized by a high incidence of externalizing problem behaviors, driven by the developmental challenges and interpersonal conflicts that adolescents face. Specific examples of these behaviors include increased risky behaviors such as smoking, drinking, fighting, truancy, and other delinquent activities (4). The immediate and long-term consequences of these behaviors are profound, as they not only disrupt adolescents’ current academic and social interactions (5) but also adversely affect their future social integration and well-being (6). Therefore, this study aims to examine the factors influencing adolescents’ externalizing problem behaviors, which is crucial for effective prevention and intervention. Although internalizing behaviors are also significant, this research focuses on externalizing behaviors due to their greater social harm and their higher likelihood of escalation into criminal activities.

Since the 19th century, physical exercise has been employed as a means of social control over adolescents. In recent years, it has been recognized as an effective method for promoting adolescent behaviors (7). Substantial evidence suggests that physical exercise not only enhances adolescents’ physical fitness and cognitive abilities (8) but also improves intrinsic values such as self-esteem (9) and self-control (10). Therefore, could physical exercise serve as a strategy to reduce adolescents’ externalizing problem behaviors? Although some studies suggest that physical exercise reduces externalizing problem behaviors, there are also opposing views that it may spawn risky behaviors, potentially increasing externalizing problem behaviors (11–14).

The question of whether physical exercise reduces adolescents’ problem behaviors has not been consistently empirically supported, due to several limitations. First, previous studies have inadequately considered the potential endogeneity in the relationship between physical exercise and externalizing problem behaviors. Second, they have not thoroughly explored the differences in the effects of physical exercise on externalizing problem behaviors across various groups. Third, the specific mechanisms through which physical exercise affects adolescents’ externalizing problem behaviors remain unclear. Although Zhang and Qian (15) have explored the pathways through which physical exercise influences deviant behavior from the perspectives of sleep quality and mental health, research suggests that self-esteem, self-control, and parent–child relationships are also important factors influencing adolescents’ externalizing problem behaviors (16–18). These factors are deserving of further investigation. To fill these gaps, this study employs the propensity score matching method to reduce selection bias and uses quantile regression analysis to explore the differences in the effects of physical exercise in adolescents with varying levels of externalizing problem behaviors, as well as subgroup regression analysis to examine differences based on gender, residential location, and family economic status. Additionally, this study also examines the mediating roles of self-esteem, self-control, and parent–child relationships in the relationship between physical exercise and externalizing problem behaviors among Chinese adolescents. The aim is to provide a scientific basis for the formulation and implementation of relevant policies and practices.

### 1.1 Effects of physical exercise on adolescents’ externalizing problem behaviors

Social Bond Theory posits that individual behavior is profoundly influenced by the social environment, in which interpersonal relationships and social identity significantly influence decision-making and behavior (19). Sports as a social activity can provide an effective platform for youth to enhance social connections and interactions. Existing research has shown that through interactions with coaches, teammates, and other athletes, adolescents receive emotional, informational, and substantive support, which, in turn, enhances their sense of belonging and commitment. This sense of social connection plays an important role as a regulator of adolescents’ externalizing problem behaviors (20). Additionally, physical exercise fosters rule adherence, as adolescents who participate in sports are required to follow specific rules, disciplines, and training schedules. This helps them internalize positive behavioral norms, effectively preventing externalizing problem behaviors (21).

Empirical research further confirms the negative impact of sports on adolescents’ externalizing problem behaviors. For example, Zhang (22) observed a negative correlation between sports and deviant behaviors in a sample of 8,670 students; longitudinal studies have also documented similar trends. One such study found that adolescents who regularly participated in team sports and endurance sports experienced reductions in tobacco and cannabis use in adulthood (23). Additionally, Brosnan (24), in an analysis of the impact of sports participation on crime across 323 local authorities in the United Kingdom between 2012 and 2015, determined that a 10% increase in sports participation led to a 0.65% decrease in property crime.

The influence of physical exercise on adolescents’ externalizing problem behaviors may be moderated by gender. According to social learning theory, gender roles significantly shape individual behavior. Social expectations often prescribe that men should demonstrate competitiveness and independence, whereas women are encouraged to develop nurturing and dependent traits (25). In the context of physical exercise, men often exhibit stronger competitiveness and ambition, which leads to greater aggression and impulsivity in groups that regularly engage in exercise, compared to women (26, 27). Additionally, gender differences in sport preferences may also influence the moderating effect of physical exercise on externalizing problem behaviors. Research indicates that contact sports preferred by men (such as football, rugby, and hockey) are often associated with higher levels of aggressive behavior (28), whereas non-contact sports preferred by women (such as tennis and golf) are with reduced aggression and other externalizing problem behaviors (29). Physiological differences are also key factors in the moderating effect of gender. For instance, the association between higher testosterone levels and aggressive behavior in men (30) may amplify the differential impact of physical exercise across genders. Therefore, while physical exercise generally helps reduce adolescents’ externalizing problem behaviors, this effect may be more pronounced in women due to men’s more competitive social role expectations and physiological differences. This suggests that gender is not only a significant factor influencing externalizing problem behaviors but also plays a crucial moderating role in the relationship between physical exercise and these behaviors. Based on this analysis, the study formulates the following hypotheses:

*H*1: Physical exercise can significantly reduce adolescents’ externalizing problem behaviors.

*H*2: Gender moderates the relationship between physical exercise and adolescents’ externalizing problem behaviors.

### 1.2 The mediating role of parent–child relationships

For individuals, although the primary subjective goal of physical exercise is to improve health, it also creates an important opportunity for social interaction. In group activities, participants often engage in extensive and meaningful interactions. Studies have found that regular sports participants typically exhibit higher levels of social participation and social integration (31). Specifically for adolescents, due to safety and skill limitations, they usually require parental or family accompaniment during physical exercise. To support their children in exercise tasks, parents frequently participate in these activities; in this process, they share relevant knowledge and experience, which fosters close and supportive parent–child relationships (32). Additionally, the enjoyable and competitive nature of physical exercise enriches parent–child interactions and strengthens emotional bonds. Research indicates that adolescent physical exercise is conducive to emotional connections between parents and children, giving a strong boost to parent–child relationships (33, 34).

Furthermore, interactions with parents have a profound impact on adolescent behavioral development. Studies have found that close and harmonious parent–child relationships enhance self-regulation in adolescents and reduce oppositional defiant disorder and other behavioral issues (35). Through long-term verbal interaction and emotional communication, parents establish deep emotional bonds with their children and promote adolescents’ moral identity through warm, accepting, and supportive parenting practices. This moral identity helps adolescents internalize social norms and develop healthy values and behavioral patterns (36). Positive parent–child relationships not only support adolescents in their self-worth recognition and receipt of care from others but also enhance their confidence and ability to cope with adversity, thus enabling their effective management of emotional stress and interpersonal conflicts during adolescence (37). Conversely, poor parent–child relationships may deplete adolescents’ psychological resources, thereby elevating the risk of antisocial behaviors (38). Moreover, the quality of parent–child relationships can indirectly predict adolescents’ capacity for interpersonal relationship management. The tendency of adolescents brought up in positive parent–child relationships to maintain more positive interactions in social and school environments lowers the likelihood of externalizing problem behaviors (39). Therefore, positive parent–child relationships not only improve adolescents’ behavioral norms and moral values but also lay a foundation for sound development. Based on the above analysis, the study formulates the following hypothesis:

*H*3: Parent–child relationships mediate the relationship between physical exercise and externalizing problem behaviors.

### 1.3 The mediating role of self-esteem

Numerous studies have revealed the critical role of self-esteem in adolescents’ mental health and behavioral development. Specifically, high self-esteem is strongly associated with reduced externalizing problem behaviors (18), better mental health, greater well-being, and improved adaptability (40). Rosenberg (41) further illustrated that low self-esteem weakens adolescents’ connection to society and reduces their identification with social norms, which may lead to an increase in delinquent behaviors. Low self-esteem also gives rise to excessive sensitivity and vulnerability to criticism and rejection, inducing depression, anxiety, and social adjustment problems (42, 43). Further research has shown that enhancement in adolescents’ self-esteem can significantly reduce such problem behaviors (44), indicating the importance of increased self-esteem in the prevention of externalizing problem behaviors and the promotion of positive adolescent growth.

Physical exercise, as a positive physical and mental activity, significantly impacts the development of self-esteem in adolescents. Studies have shown that participation in sports greatly enhances adolescents’ psychological health and social adaptability. Specifically, physical exercise directly boosts adolescents’ self-esteem by improving body image and self-efficacy (45). Moreover, group exercise strengthens social ties among adolescents and indirectly enhances self-esteem by increasing their sense of belonging and social identity through teamwork and the pursuit of common goals (46). In addition, success and challenge overcoming in sports competition provide adolescents with important opportunities to validate their self-worth and competence, further strengthening their self-esteem (47). Based on the above analysis, the study formulates the following hypothesis:

*H*4: Self-esteem mediates the relationship between physical exercise and externalizing problem behaviors.

### 1.4 The mediating role of self-control

Self-control is defined as an individual’s ability to adjust their thoughts, feelings, and behaviors promptly to achieve a set goal when the external environment changes (48). This ability is widely considered to be a key factor influencing adolescents’ externalizing problem behaviors (49). A large body of research supports that individuals with lower self-control are more likely to exhibit externalizing problem behaviors such as aggression, ADHD, Internet addiction, and delinquency (17, 50–52).

Studies have shown that regular sports participants have greater self-control compared to those physically inactive (53), suggesting a positive association between physical exercise and improved self-control. Moreover, different durations (54, 55) and intensities (56, 57) of exercise have been shown to enhance self-control. The relationship between physical exercise and the enhancement of executive functions in the brain provides a biological basis for its effects on self-control. Executive functions, including planning, inhibitory control, and working memory, are important factors influencing self-control (58). Studies have shown that physical exercise stimulates activity in brain regions associated with executive functions. As observed through functional magnetic resonance imaging (fMRI), physical exercise can lead to positive changes in the cognitive control system of the brain (54). Based on the above analysis, the study formulates the following hypothesis:

*H*5: Self-control mediates the relationship between physical exercise and externalizing problem behaviors.

## 2 Materials and methods

### 2.1 Data source and study population

The data for this study come from the China Family Panel Studies (CFPS),^1^ a program conducted under the auspices of the Institute of Social Sciences at Peking University. The CFPS sample covers 25 provinces, municipalities, and autonomous regions in China, representing 95% of the national population. Therefore, the CFPS can be regarded as a nationally representative sample (59). This study used data from the 2018 CFPS, which included 2,620 participants aged 10 to 15. After excluding samples with critical missing values, the final valid sample consisted of 2,468 participants. The investigation was reviewed and approved by the Biomedical Ethics Committee of Peking University (approval number: IRB00001052-14010) and was conducted in accordance with the guidelines of the Declaration of Helsinki and its amendments. Written informed consent was obtained from both participants and their guardians prior to their participation in this study.

### 2.2 Variables

#### 2.2.1 Externalizing problem behaviors

The 2018 CFPS introduced the collection of data on externalizing problem behaviors for the first time in adolescents aged 10–15 years. The 2018 CFPS Externalizing Problem Behaviors Scale (EPBS) was adapted from a condensed version of the Early Childhood Longitudinal Study in the United States (60). The scale consists of six items: “I often argue with my peers,” “It is hard for me to pay attention,” “I get distracted easily,” “It is hard for me to finish my school work,” “I get in trouble for talking and disturbing others,” and “I get in trouble for fighting with my peers.” However, the items “It is hard for me to pay attention” and “I get distracted easily” conceptually overlap with the mediating variable of self-control, which may result in circular reasoning during the analysis. Additionally, externalizing problem behaviors should mainly reflect the frequency of problematic actions, while these two items place more emphasis on evaluating self-control. Thus, to avoid measurement ambiguity and improve the scale’s accuracy, these two items were excluded from the study.

Ultimately, the scale retained four items, with a scoring range from 1 to 5, where 1 represents “completely inconsistent” and 5 signifies “completely consistent.” Summing the scores of these four items yields the total score for adolescent externalizing problem behaviors; a higher total score reflects greater severity of such behaviors. In this study, the Cronbach’s alpha coefficient of the scale was 0.67, suggesting an acceptable level of internal consistency.

#### 2.2.2 Physical exercise

The CFPS survey assesses physical exercise through two questions: “How often did you exercise in the last week?” and “How long did you exercise in the last week?” In this study, responses indicating “more than 360 min of exercise per session” were excluded as outliers. Subsequently, the average daily duration of physical exercise among adolescents was calculated using the formula: average daily exercise time (in minutes) = weekly exercise time / 7. To better align the independent variable with normal distribution requirements and to ensure that samples with an average daily exercise time of zero were not excluded, 1 min was added to the average daily exercise time before taking the natural logarithm (61, 62). This results in a continuous variable that measures the physical exercise level of adolescents; the higher the value, the greater the level of physical exercise.

#### 2.2.3 Parent–child relationships

According to existing research, parent–child relationships are measured through the emotional connection between parents and their children (63). Specifically, adolescents were asked about the number of quarrels they had with their parents per week and the number of heart-to-hearts talks they had; the responses to both questions ranged from 0 to 13.5 (calculated based on monthly occurrences). Whether parents know who the adolescent is with when they are not at home is another major indicator, as measured by the question “What percentage of parents know who you are with when you are not at home?” Responses ranged from 1 (“Always know”) to 5 (“Never know”). Additionally, the CFPS questionnaire asked respondents to rate their trust in their parents on a scale from 1 (“very distrustful”) to 10 (“very trusting”). To calculate parent–child relationship scores, the responses to “the number of weekly quarrels with parents” and “how much parents know who you are with when you are not at home” were reverse-coded. These reverse scores, along with the other two indicators, were summed to derive a composite measure of parent–child relationships. A higher score indicates a stronger emotional connection between parents and children.

#### 2.2.4 Self-esteem

In the CFPS, respondents’ self-esteem was measured on the Self-Esteem Scale (RSES) developed by Rosenberg (41), which consists of 10 items: (1) I am no worse than anyone else; (2) I have many good qualities; (3) I am a failure; (4) I can do things well; (5) There is not much for me to be proud of; (6) I am sure of myself; (7) I am satisfied with myself; (8) I want to earn respect; (9) I am useless; (10) I consider myself good for nothing. Each item is rated on a scale from 1 (“disagree completely”) to 5 (“agree completely”). To calculate an individual self-esteem score, the five negative worded items (3), (5), (8), (9), and (10) were reverse-scored, and then all items were summed to derive an overall self-esteem score, with higher scores indicating higher self-esteem. Missing individual data were replaced by the previous data. In this study, the Cronbach’s alpha coefficient was 0.705, indicating good reliability.

#### 2.2.5 Self-control

The 2018 CFPS survey assessed self-control on the Self-Control Scale (SCS), which comprises 12 items: (1) I am always well-prepared; (2) I pay close attention to details; (3) I like to be organized; (4) I will do things according to my own schedule; (5) I am very careful in my studies; (6) I always place things in a random manner; (7) I always mess things up; (8) I always forget to restore things; (9) I am careful and thorough; (10) I do my homework before I play; (11) I start my homework as soon as it is set; (12) I clean up after things are messed up (64). Each item is rated on a 1–5 scale, from 1 (disagree completely) to 5 (agree completely). To calculate the individual self-control score, the three negative items (6), (7), and (8) are reverse-scored, with all items then summed to derive an overall self-control score; higher scores indicate stronger self-control. Missing individual data were replaced by the previous data. In this study, the Cronbach’s alpha coefficient was 0.791, indicating good reliability.

#### 2.2.6 Control variables

In this study, control variables were selected based on three dimensions: individual, family, and school characteristics. For individual characteristics, commonly used demographic characteristics were included, such as adolescents’ age, gender, residence, and education level. Additionally, studies indicate that academic pressure and psychological conditions, such as depression, significantly affect adolescents’ externalizing problem behaviors (65, 66). Therefore, the control variables for individual characteristics were: age, gender (male = 1, female = 0), residence (urban = 1, rural = 0), education level (1 = elementary school, 2 = junior secondary school, 3 = senior secondary school), academic pressure (rated on a 1–5 scale, from no pressure to a lot of pressure), and depression (measured on the CES-D scale, with scores ranging from 22 to 72, where higher scores indicate more severe depression). Studies highlight the importance of family characteristics, including parental education level and family economic status, for adolescent behavioral development (67). Therefore, the control variables for family characteristics included parental education level (1 = elementary school or below, 2 = secondary school, 3 = university or above) and family economic status (measured by quartiles of annual household income). School-related factors also have a significant impact on adolescents’ externalizing problem behaviors. Factors such as public school status, key school status, and class type are usually related to the quality of education students receive and may influence adolescents’ behavior (68, 69). Therefore, the control variables for school characteristics were: public school status (1 = public school, 0 = private school), key school status (1 = key school, 0 = non-key school), and class type (1 = non-key class, 2 = no distinction, 3 = key class, treated as a dummy variable in subsequent analysis).

In summary, Table 1 presents the descriptive statistics for the key variables examined in this study.

**Table 1:** The definition of variables and descriptive statistical results.

Variable name	Variables definition	Mean	SD	N
Externalizing problem behaviors	Externalizing problem behaviors test scores	8.466	2.666	2,442
Physical exercise	The logarithm of the average daily Physical exercise time	2.339	1.749	2,468
Parent–child relationship	Parent–child relationship scores	26.669	2.403	2,449
Self-esteem	Self-Esteem test scores	35.791	4.007	2,451
Self-control	Self-control test scores	42.737	6.697	2,342
Age	Age	12.408	1.684	2,468
Gender	Male =1, Female =0	0.535	0.499	2,468
Residence	Urban =1, Rural =0	0.413	0.493	2,459
Education level	1 = Elementary school, 2 = Junior Secondary School, 3 = Senior Secondary School	1.426	0.530	2,442
Academic pressure	Academic pressure test scores	2.897	1.138	2,440
Depression	CES-D test scores	29.942	6.246	2,465
Parental education level	1 = Elementary school or below 2 = Secondary school 3 = University or above	1.865	0.631	2,468
Family economic status	Annual household income (quartiles)	2.503	1.062	2,445
Public School Status	1 = Public school, 0 = Private school	0.904	0.294	2,406
Key School Status	1 = key school, 0 = non-key school	0.225	0.418	2,368
Type of class	1 = non-key class, 2 = No distinction between key and non-key classes, 3 = key class	1.901	0.569	2,438
Physical exercise status	1 = Regular physical exercise 0 = Infrequent physical exercise	0.522	0.500	2,468

### 2.3 Methodology specification

(1) The impact of physical exercise on externalizing problem behaviors was first assessed using Ordinary Least Squares (OLS) with the following equation:

(1) $$EPB_{i}=a+\beta_{1}Exercise_{i}+\beta_{2}Control_{i}+\in_{i}$$

In Equation 1, EPB_i_ represents the externalizing problem behaviors score of individual i, Exercise_i_ is the logarithm of physical exercise time of individual i, Control_i_ represents the control variable, a is the intercept term, β_1_ and β_2_ are the coefficients of the physical exercise time variable and the control variable, respectively, and ε_i_ is the random error term.

(2) To confirm the robustness of the OLS model results and reduce potential selection bias, this study further employed the Propensity Score Matching (PSM) model. This method assesses the Average Treatment Effect of Treated (ATT) of physical exercise on externalizing problem behaviors using the following equation:

(2) $$ATT=E\left\{E\left[EPB_{1i}|D_{i}=1,p\left(x_{i}\right)\right]-E\left[EPB_{0i}|D_{i}=0,p\left(x_{i}\right)\right]\right\}$$

In Equation 2, EPB_1i_ and EPB_0i_ represent the scores of externalizing problem behaviors of adolescents who regularly engage in physical exercise and adolescents who infrequently engage in physical exercise, respectively. D_i_ = 1 when individual i participates in physical exercise regularly, and vice versa D_i_ = 0. p(X_i_) is the probability that individual i engages in physical exercise.

(3) To investigate the heterogeneity in the impact of physical exercise on externalizing problem behaviors across various levels, this study utilized quantile regression analysis. This method enabled a detailed analysis of the influence of explanatory variables on outcome variables across different quartiles, thereby offering a comprehensive understanding of how physical exercise influences adolescents’ behavioral patterns.

(3) $$Quantile_{\tau }\left(CA\right)=a_{\tau }+\beta_{1\tau }Exercise_{i}+\beta_{2\tau }Control_{i}+\in_{i\tau }$$

In Equation 3, a _T_, β_1T_, β_2T_ and ε_iT_ denote the intercept at the Tth quantile, the coefficients of the physical exercise time variables, the coefficients of the control variables, and the random error terms, respectively.

(4) The study further analyzed the heterogeneity of the impact of physical exercise on adolescents’ externalizing problem behaviors, stratified by gender, residence, and family economic status.
(5) Finally, this study employed Model 4 of the PROCESS macro by Hayes to assess the mediating roles of parent–child relationships, self-esteem, and self-control in the link between physical exercise and adolescents’ externalizing problem behaviors. The analysis used 5,000 bootstrap samples, and effects were deemed significant when the 95% confidence intervals did not include zero.

## 3 Results

### 3.1 The relationship between physical exercise and adolescents’ externalizing problem behaviors

Table 2 presents the estimation results from the multivariate linear model. Model 1, which includes only the core explanatory variable (physical exercise) and control variables for individual adolescent characteristics, showed a physical exercise coefficient of −0.096, which could significantly and negatively predict externalizing problem behaviors (*p* < 0.01). In Model 2, the inclusion of control variable for family characteristics results in a slight increase in the physical exercise coefficient to −0.088 (*p* < 0.01). Model 3, which further controls for school characteristics, reveals a decrease in the physical exercise coefficient to −0.095 (*p* < 0.01).

**Table 2:** The relationship between physical exercise and adolescents’ externalizing problem behaviors: results of OLS.

Variables	Model (1)	Model (2)	Model (3)
Physical exercise	−0.096*** (0.029)	−0.088*** (0.029)	−0.095*** (0.030)
Individual characteristics	Control	Control	Control
Family characteristics		Control	Control
School characteristics			Control
Constant	3.844*** (0.508)	4.456*** (0.564)	4.371*** (0.615)
N	2,428	2,405	2,331
R^2^	0.144	0.148	0.149

Confidence level: 95%; *p < 0.1, **p < 0.05, ***p < 0.01 (two-tailed). Standard errors are presented in parentheses.

### 3.2 Heterogeneity in the effects of physical exercise on adolescents’ externalizing problem behaviors

#### 3.2.1 Robustness tests for the role of physical exercise: estimation based on the PSM

Given the implications of survey data limitations, variable control, and sample selection bias, the current study additionally employed the PSM model to estimate the net effect of physical exercise on externalizing problem behaviors.

In this study, adolescents who engaged in physical exercise at least 3 days a week were considered to be “regular physical exercisers” (treatment group), while those who failed to meet this criterion were considered to be “physically inactive” (control group). Physical exercise was coded as a dummy variable (1 for the treatment group, 0 for the control group) to meet the requirements of the propensity score matching method. The matching results indicated that the absolute values of all standardized deviations after matching were less than 10%. Additionally, the double t-distribution test also indicated no significant differences between all the variables after matching (*p* > 0.1), suggesting that sample bias was largely addressed through PSM.

To accurately estimate the Average Treatment Effect of Treated (ATT) of regular physical exercise on adolescents’ externalizing problem behaviors, the present study employed three methods of propensity score matching: nearest-neighbor matching (*k* = 3), nearest-neighbor matching with caliper (*k* = 3; *r* = 0.01), and kernel matching (default kernel functions and bandwidths; see Table 3). The ATT estimates for physical exercise were − 0.361 (*p* < 0.01), −0.362 (*p* < 0.01), and − 0.323 (*p* < 0.01) under the three matching strategies, suggesting that adolescents who regularly engaged in physical exercise (at least three times per week) demonstrated reductions in their externalizing problem behavior scores of 0.361, 0.362, and 0.323, respectively, compared to their less active peers. After comparing the ATT with the parameter estimate of Model (3) from the OLS regression (0.095), the heightened effect of physical exercise on externalizing behaviors, as estimated by the PSM method, can be attributed to the sample grouping criteria employed. This, in turn, explains the reliability of the treatment and control group classifications used in this study. Therefore, Hypothesis 1 has been fully validated.

**Table 3:** The relationship between physical exercise and adolescents’ externalizing problem behaviors: results of PSM estimation.

Matching method	T	C	ATT	Standard errors
Nearest-neighbor matching	8.313	8.674	−0.361***	0.127
Nearest-neighbor matching with caliper	8.313	8.675	−0.362***	0.127
kernel matching	8.315	8.638	−0.323***	0.113

Confidence level: 95%; *p < 0.1, **p < 0.05, ***p < 0.01 (two-tailed).

#### 3.2.2 The relationship between physical exercise and adolescents’ externalizing problem behaviors: the effect of the degree of individual externalizing problem behaviors

OLS regression quantifies the average effect of physical exercise on externalizing problem behaviors. In contrast, quantile regression more effectively illustrates how these effects vary across different levels of the response variable. In this study, quantiles of 0.1, 0.25, 0.5, 0.75, and 0.9 were selected to represent low, medium-low, medium, medium-high, and high levels of adolescents’ externalizing problem behaviors, respectively. The results indicated that parameter estimates at the 0.1, 0.25, 0.5, 0.75, and 0.9 quantiles were − 0.120 (*p* < 0.01), −0.112 (*p* < 0.01), −0.094 (*p* < 0.05), −0.086 (*p* < 0.05), and − 0.081 (*p* < 0.1), respectively (see Table 4). Overall, the inhibitory impact of physical exercise on externalizing problem behaviors exhibited a decreasing trend as the severity of these behaviors increased.

**Table 4:** Quantile regression of physical exercise on adolescents’ externalizing problem behaviors.

Variables	Model (4)	Model (5)	Model (6)	Model (7)	Model (8)
	τ = 0.1	τ = 0.25	τ = 0.5	*τ* = 0.75	τ = 0.9
Physical exercise	−0.120***	−0.112***	−0.094**	−0.086**	−0.081*
	(0.038)	(0.041)	(0.040)	(0.040)	(0.046)
Individual characteristics	Control	Control	Control	Control	Control
Family characteristics	Control	Control	Control	Control	Control
School characteristics	Control	Control	Control	Control	Control
Constant	0.791	2.339***	3.473***	5.442***	8.623***
	(0.632)	(0.850)	(0.781)	(0.933)	(1.143)
N	2,331	2,331	2,331	2,331	2,331
R^2^	0.069	0.065	0.073	0.099	0.114

Confidence level: 95%; *p < 0.1, **p < 0.05, ***p < 0.01 (two-tailed). Standard errors are presented in parentheses.

**Figure 1:**
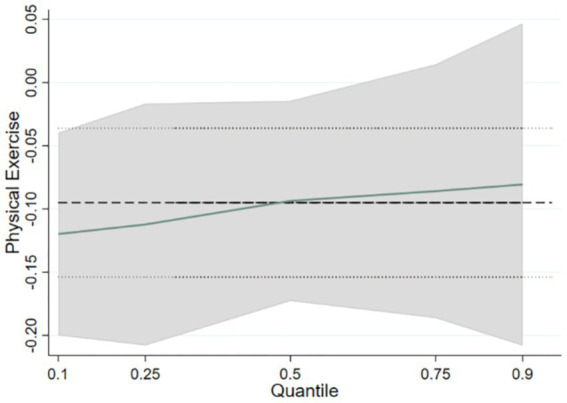
Changes in physical exercise at different percentiles of externalizing problem behavior.

The plot of quantile coefficient changes (see Figure 1) illustrates the trend of physical exercise coefficients at different quantile points. The dashed line denotes the OLS regression estimates of the explanatory variables, the solid line denotes the quantile regression estimates, and the gray shading illustrates the 95% confidence interval for the quantile regression estimates. Analysis reveals that the negative effect of physical exercise on externalizing problem behaviors diminishes with the increase in the severity of these behaviors. The inhibitory effect falls below the OLS regression estimates starting at the 0.5 quantiles and beyond, and it only reaches the 10% statistical significance threshold at the 0.9 quantiles. This indicates that the negative impact of physical exercise is pronounced for adolescents with fewer externalizing problem behaviors; for those with more externalizing problem behaviors, the inhibitory effect weakens and becomes less significant.

#### 3.2.3 The relationship between physical exercise and adolescents’ externalizing problem behaviors: the effects of gender, residence, and family economic status

This study further explored the heterogeneity of the effects of physical exercise on externalizing problem behaviors by categorizing adolescents based on gender, place of residence, and family economic status (low-income in the bottom 50% and high-income in the top 50% of the household income quartile). Firstly, from a gender perspective, the negative impact of physical exercise on externalizing problem behaviors was significantly more pronounced for females compared to males. For males, this effect was statistically significant only at the 10% level (Table 5). Therefore, Hypothesis 2 is partially supported. Secondly, with regard to the place of residence, the negative impact of physical exercise was significantly stronger among urban adolescents than rural adolescents. For rural adolescents, this effect was not statistically significant. Regarding family economic status, physical exercise exerts a significant inhibitory effect on externalizing problem behaviors in both economically advantaged and disadvantaged adolescents, with a significantly more substantial impact on economically advantaged adolescents compared to their disadvantaged counterparts.

**Table 5:** Effects of physical exercise on adolescents’ externalizing problem behaviors by gender, household registration, and family economic status.

Variables	Problem behaviors					
	Gender	Residence			Family economic status	
	Model (9)	Model (10)	Model (11)	Model (12)	Model (13)	Model (14)
	Male	Female	Urban	Rural	Low income	High income
Physical exercise	−0.077* (0.043)	−0.113*** (0.042)	−0.134*** (0.046)	−0.065 (0.040)	−0.080* (0.042)	−0.115*** (0.043)
Individual characteristics	Control	Control	Control	Control	Control	Control
Family characteristics	Control	Control	Control	Control	Control	Control
School characteristics	Control	Control	Control	Control	Control	Control
Constant	5.196*** (0.879)	4.498*** (0.839)	4.449*** (0.959)	4.214*** (0.816)	4.916*** (0.870)	4.073*** (1.002)
N	1,258	1,073	966	1,365	1,204	1,127
R^2^	0.105	0.181	0.173	0.122	0.134	0.159

Confidence level: 95%; *p < 0.1, **p < 0.05, ***p < 0.01 (two-tailed). Standard errors are presented in parentheses.

*Post-hoc* comparisons (Supplementary Table S1) revealed that externalizing problem behaviors were more frequent among males, rural adolescents, and adolescents from economically disadvantaged families compared to females, urban adolescents, and adolescents from economically advantaged families. The results of the post-hoc comparisons, together with those in Table 5, corroborate the findings from the quantile regression analysis: for adolescents with fewer externalizing problem behaviors, the negative impact of physical exercise is pronounced; however, for those with more pronounced externalizing problem behaviors, this effect diminishes and becomes less evident. This suggests that physical exercise effectively mitigates early externalizing problem behaviors; however, more severe cases may require an additional multifaceted intervention.

### 3.3 Analysis of the mediating role of parent–child relationships, self-esteem and self-control

**Figure 2:**
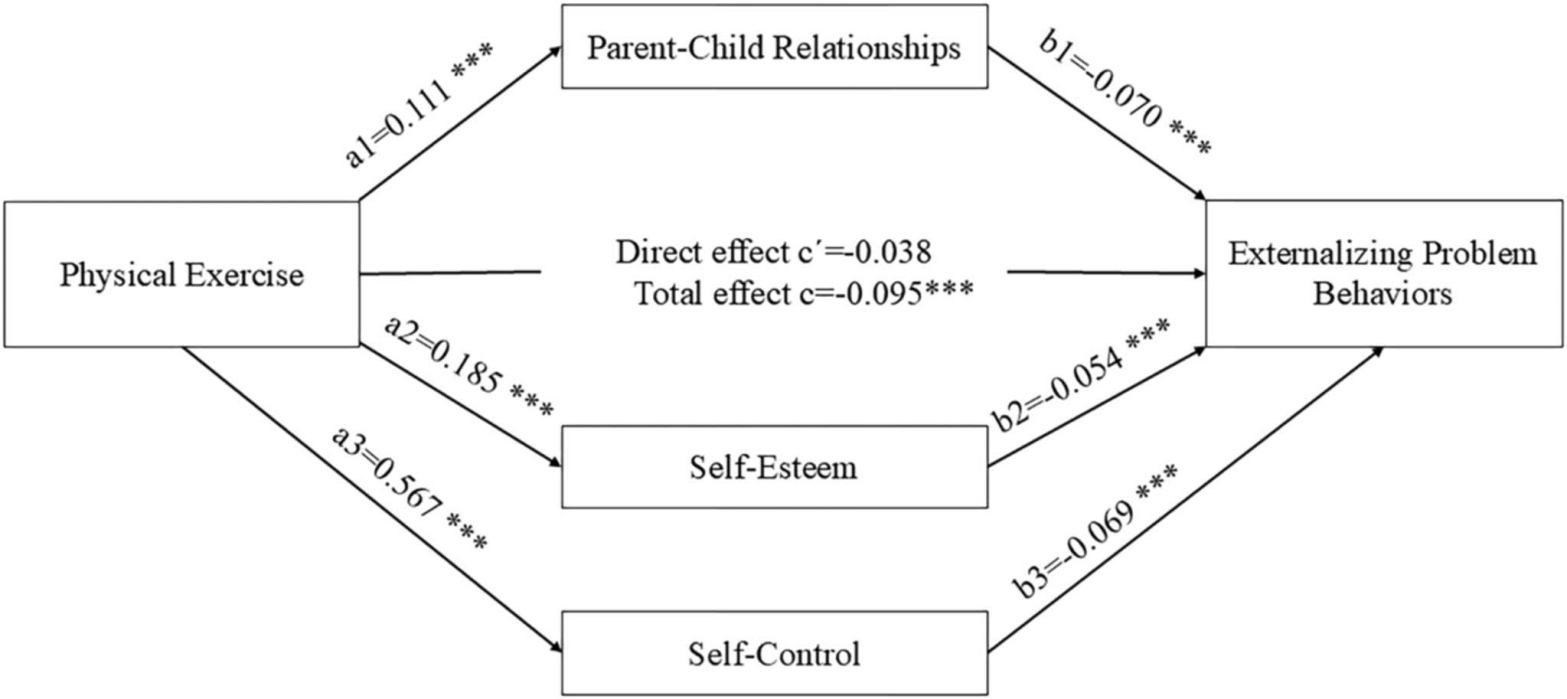
Mediation model of parent–child relationships, self-esteem, and self-control as mediators between physical exercise and externalizing problem behaviors ***p < 0.01,**p < 0.05,*p < 0.1, the mediating effect of parent–child relationships on the relationship between physical exercise and externalizing problem behaviors was a1*b1; the mediating effect of self-esteem was a2*b2; the mediating effect of self-control was a3*b3; the direct effect of physical exercise on externalizing problem behaviors was cˊ; and the total effect of physical exercise on externalizing problem behaviors was c.

According to the theoretical analysis, emotional ties between adolescents and their parents, as well as self-esteem and self-control, may mediate the relationship between physical exercise and externalizing problem behaviors. Consequently, bootstrap mediation analysis was employed to test the mediating effects. The results indicated that the total effect of physical exercise on adolescents’ externalizing problem behaviors was significant when controlling for covariates (CI: −0.156; −0.034). Although the direct effect of physical exercise on externalizing problem behaviors was not significant (CI: −0.098; 0.022), physical exercise was found to have indirect effects through three mediating pathways: physical exercise → parent–child relationships → externalizing problem behaviors (CI: −0.015; −0.002), physical exercise → self-esteem → externalizing problem behaviors (CI: −0.019; −0.003) and physical exercise → self-control → externalizing problem behaviors (CI: −0.055; −0.025). These findings suggest that parent–child relationships, self-esteem, and self-control played a fully mediating role in the relationship between physical exercise and externalizing problem behaviors. Consequently, Hypotheses 3, 4, and 5 were confirmed. The results are displayed in Table 6 and Figure 2.

**Table 6:** Analysis of the mediating role of parent–child relationships, self-esteem and self-control.

Effect	Estimate	BootSE	BootLLCI	BootULCI
Total effect (physical exercise)	−0.095	0.031	−0.156	−0.034
Direct effect (physical exercise)	−0.038	0.031	−0.098	0.022
Indirect effect (parent–child relationships)	−0.008	0.003	−0.015	−0.002
Indirect effect (self-esteem)	−0.010	0.004	−0.019	−0.003
Indirect effect (self-control)	−0.039	0.008	−0.055	−0.025

*N* = 2,198, BootSE represents the standard error, BootLLCI and BootULCI denote the lower and upper bounds, respectively, of the indirect effect within the 95% confidence interval, as estimated by the bias-corrected percentile Bootstrap method.

## 4 Discussion

### 4.1 Effects of physical exercise on adolescents’ externalizing problem behaviors

The findings of the study demonstrate that physical exercise significantly impacts adolescents’ externalizing problem behaviors, even after controlling for individual, family, and school characteristics. For every 1% increase in the duration of physical exercise, there is a corresponding decrease of 0.095 points in the score of externalizing problem behaviors, amounting to 1.122% of the average score for these behaviors. To address potential selection bias, this paper employed the PSM method, confirming the robustness of the negative impact of physical exercise on adolescents’ externalizing problem behaviors. These results underscore the critical role of physical exercise in adolescent development and advocate its use as an effective intervention to mitigate adolescents’ externalizing problem behaviors.

Furthermore, the study revealed heterogeneity in the effects of physical exercise on adolescents’ externalizing problem behaviors based on gender, residence, and family economic status. Specifically, physical exercise is more effective for females than for males in reducing externalizing problem behaviors. This effect was more pronounced among urban adolescents than among rural adolescents. Additionally, adolescents from more affluent families benefited more from physical exercise than those from less affluent families. Moreover, quantile regression analyses further indicated that physical exercise was more effective in reducing externalizing problem behaviors in adolescents with fewer such behaviors.

Social learning theory serves as a robust theoretical foundation for understanding the gender differences in the impact of physical exercise on externalizing problem behaviors. Specifically, males and females are subject to distinct role expectations in behavioral norms and the socialization process. Males are typically encouraged to demonstrate competitiveness and autonomy, while females are guided to cultivate traits like care and dependence (25, 70). This difference in gender roles is especially evident in sports activities, where males often display a stronger desire for competition, which may be associated with increased aggressive behavior (71). Moreover, there are significant differences between males and females in their choice of sports. Males are more inclined to sports focused on strength and speed with high levels of physical confrontation, whereas females generally prefer non-contact sports characterized by flexibility or balance (72). Research has shown that participation in contact sports, such as football, does not necessarily reduce aggressive behavior. In contrast, non-contact sports, such as tennis, are associated with a significant decrease in externalizing problem behaviors (73). Biological differences also account for this phenomenon. Studies have shown that testosterone levels in males increase after exercise, and testosterone is linked to aggressive and other externalizing behaviors (74). Therefore, the combined effects of the socialization process and biological differences increase the effectiveness of physical exercise in the mitigation of externalizing problem behaviors in female adolescents compared to males.

Existing literature suggests that externalizing problem behaviors are generally higher among male adolescents than female ones, among rural adolescents compared to urban adolescents, and among adolescents from less well-off families (75). The results of the present study support these views (Supplementary Table S1) and further emphasize the importance of physical exercise in preventing adolescents’ externalizing problem behaviors through quantile regression analysis. In particular, for adolescents with fewer externalizing problem behaviors, physical exercise can be an effective intervention to reduce or even prevent such behaviors. However, for adolescents with more significant externalizing problem behaviors, physical exercise alone may not produce significant improvements. Therefore, a combination of other interventions is recommended. The promotion of physical exercise should be integrated into adolescents’ health development strategies, especially before the escalation of externalizing problem behaviors. Active participation in physical exercise can help prevent and mitigate these behaviors.

This study investigated the mediating pathways through which physical exercise affects adolescents’ externalizing problem behaviors. Zhang and Qian (15) similarly examined these pathways and found that sleep quality and mental health mediate the relationship between physical exercise and deviant behavior. However, their study failed to include control variables, which may have led to endogenous bias in their results. In response, this present study introduced depression, an important dimension of mental health, as a control variable and selected a comprehensive set of control variables at the individual, family, and school levels. Simultaneously, the propensity score matching method was employed in this study to mitigate selection bias and, thereby, address endogenous issues that previous studies may not have adequately solved. The findings reveal that self-esteem, self-control, and parent–child relationships are important mediators between physical exercise and adolescents’ externalizing problem behaviors. This provides new empirical evidence for research on the complex mechanisms between physical exercise and externalizing problem behaviors.

### 4.2 The mediating role of parent–child relationships

The results of this study clearly indicate that physical exercise can significantly reduce adolescents’ externalizing problem behaviors, with the improvement of parent–child relationships as a key mediating factor. First, physical exercise has a positive impact on adolescents’ self-control, social skills, and emotional regulation, which are key factors in reducing externalizing problem behaviors (17, 76). More importantly, physical exercise provides adolescents with the opportunity to participate alongside their parents, enhancing intra-family interaction and communication. Enhanced parent–child relationships provide adolescents with emotional support and a sense of identity, which contributes to positive behavioral changes. Good parent–child relationships can provide a stable emotional foundation, increasing adolescents’ resilience. Specifically, positive parent–child interactions and effective communication during joint participation in physical exercise can significantly reduce antisocial behaviors and emotional problems among adolescents (77). Therefore, the interaction between physical exercise and family factors should be comprehensively considered in the design of prevention and intervention measures for externalizing problem behaviors. Parental involvement not only enhances the benefits of physical exercise but also promotes positive behaviors in adolescents by further improving parent–child relationships.

### 4.3 The mediating role of self-esteem

Our findings strongly support the hypothesis that self-esteem mediates the relationship between physical exercise and externalizing problem behaviors. Specifically, regular physical exercise proved effective in reducing individuals’ tendency to engage in externalizing problem behaviors (e.g., smoking, drinking, and using illegal drugs) by enhancing their self-esteem. This finding is consistent with previous research, emphasizing the importance of physical exercise for psychological well-being (78) and revealing that high self-esteem may act as a protective factor against undesirable behaviors.

Self-Determination Theory and Social Identity Theory help explain these effects. Self-Determination Theory posits that the satisfaction of basic psychological needs (e.g., autonomy, competence, and relatedness) is crucial for self-esteem (79). Physical exercise provides adolescents with opportunities to demonstrate skills, achieve goals, and build social connections, all of which are vital for self-esteem. Social Identity Theory, on the other hand, suggests that individuals’ social behaviors are strongly influenced by their perceptions of self-worth (80) and that increased self-esteem enhances social adaptability and reduces perceived social exclusion, thereby decreasing externalizing problem behaviors.

The findings hold significant implications for the design of physical exercise intervention programs for adolescents. Such programs should not only focus on skill enhancement and physical fitness promotion but also integrate elements that promote self-esteem, such as achievable goals, teamwork, and positive feedback, all of which are essential for reducing adolescents’ externalizing problem behaviors.

### 4.4 The mediating role of self-control

This study investigated how physical exercise reduces adolescents’ externalizing problem behaviors by enhancing self-control. Results indicated that physical exercise significantly enhances adolescents’ self-control, which in turn effectively reduces their externalizing problem behaviors. This finding underscores the positive impact of physical exercise on adolescents’ psychological and behavioral health, particularly its mediating role in enhancing self-control. Furthermore, by enhancing self-control, physical exercise helps adolescents better manage stress and emotions, thereby diminishing externalizing problem behaviors.

Although previous research has highlighted the positive effects of both physical exercise and self-control on adolescents’ social skills and behavioral performance, the interrelationship among these factors has been relatively underexplored. This study elucidates the mediating role of self-control in the relationship between physical exercise and adolescents’ externalizing problem behaviors. According to self-control theory, greater self-control enables effective management of behaviors and emotions, thereby reducing externalizing problem behaviors (81). Physical exercise, particularly forms that require long-term commitment and self-discipline, significantly enhances self-control at both physiological and psychological levels. Physiologically, regular physical exercise enhances brain function, particularly in the prefrontal cortex, which is closely related to self-control (82). Psychologically, physical exercise offers a sound approach to the management of stress and negative emotions, which is crucial for adolescents’ self-control (83). Through physical exercise, adolescents learn to set goals, persevere, and cope with failure, an experience that promotes not only self-control but also more sensible and responsible decision-making in daily life. In summary, physical exercise constitutes an effective method for adolescents to overcome behavioral problems by enhancing self-control.

## 5 Implications, limitations and future research

The present study confirmed the effectiveness of physical exercise in suppressing adolescents’ externalizing behaviors and elucidated the mediating roles of factors such as parent–child relationships, self-esteem, and self-control. These findings offer significant insights for both theoretical research and practical application in the prevention and treatment of adolescents’ externalizing behaviors. However, this study has some limitations. First, the cross-sectional design limits the ability to infer causality from the results. Second, the study failed to not distinguish between the specific effects of different intensities and types of sports on externalizing behaviors, a notable limitation. To more deeply explore the specific effects of physical exercise, future research could collect more comprehensive data, including variations in exercise intensity and type. Additionally, longitudinal studies over several years are recommended to validate the long-term effects of physical exercise on adolescents’ externalizing behaviors.

## 6 Conclusion

This study demonstrates that physical exercise significantly inhibits adolescents’ externalizing problem behaviors. Nevertheless, the effects of physical exercise are not consistent across different populations. This inhibitory effect is especially pronounced among females, urban residents, individuals from families with better economic status, and adolescents with milder externalizing behaviors. In contrast, the effect is weaker among males, rural residents, individuals from economically disadvantaged families, and adolescents experiencing more severe externalizing behaviors. Furthermore, physical activity indirectly reduces adolescents’ externalizing problem behaviors through three mediating factors: parent–child relationships, self-esteem, and self-control.

## Data Availability

Publicly available datasets were analyzed in this study. This data can be found here: http://www.isss.pku.edu.cn/cfps/.

## Footnotes

^1^www.isss.pku.edu.cn/cfps/
