# Supplementary material for: The influence of physical exercise on adolescents’ externalizing problem behaviors: mediating effects of parent–child relationships, self-esteem, and self-control
Source: Front Public Health. 2024 Oct 25;12:1452574. doi: 10.3389/fpubh.2024.1452574 (PMC11543408; doi:10.3389/fpubh.2024.1452574)
Supplement: Supplementary file 1 [file Table_1.DOCX]

**Supplementary table S1** Post hoc comparisons results

| Variables | | Problem behaviors | T |
| --- | --- | --- | --- |
| Gender | Male | 8.793 | -6.405*** |
|  | Female | 8.060 |  |
| Residence | Urban | 8.199 | 3.784*** |
|  | Rural | 8.640 |  |
| Family economic status | Low income | 8.570 | 2.048** |
|  | High income | 8.334 |  |

***p<0.01, **p<0.05, *p<0.1
